# Does who responds matter?: exploring potential proxy response bias in the Washington Group Short Set disability estimates

**DOI:** 10.3389/frma.2025.1654769

**Published:** 2025-10-30

**Authors:** Aaron Beuoy, Kelsey S. Goddard

**Affiliations:** Institute for Health and Disability Policy Studies, University of Kansas, Lawrence, KS, United States

**Keywords:** disability estimates, proxy respondents, Washington Group Short Set (WG-SS), WG-SS and proxy respondents, WG-SS and disability estimates

## Abstract

**Introduction:**

The Washington Group Short Set (WG-SS) is a widely used tool for identifying disability in national and international population-based surveys. However, results from cognitive testing revealed key differences in response patterns between individuals who self-report and those with a proxy respondent. Considering proxy reporting is frequently used in national surveys, discrepancies between reporting sources could affect the accuracy of disability prevalence estimates and have important implications for health equity and policy.

**Methods:**

A binary logistic regression was conducted to examine the relationship between proxy respondents and WG-SS disability status after controlling for sociodemographic characteristics, using pooled data from the 2010–2018 National Health Interview Survey (NHIS).

**Results:**

After controlling for sociodemographic characteristics, proxy respondents were 4.48 times more likely to be classified as having a WG-SS disability compared to those who self-reported.

**Discussion:**

Differences in proxy reporting have real implications for equity, access, and policy accountability. If proxy reporting systematically increases the likelihood of disability classification, prevalence estimates may be distorted. This is especially problematic when proxies are more likely to report for populations already at risk of under- or overrepresentation in disability data, such as older adults, people with cognitive disabilities, and children and adolescents. Future studies using the WG-SS should treat the reporting source, i.e., proxy response, not as a procedural footnote, but as a central variable in assessing data quality and equity.

## 1 Introduction

The Washington Group Short Set (WG-SS) is one of the most widely used tools for identifying disability in population-based surveys. Developed by the Washington Group on Disability Statistics under the United Nations Statistical Commission, the WG-SS focuses on functional difficulties in six core domains—seeing, hearing, mobility, cognition, self-care, and communication—to identify individuals at risk of experiencing limitations in daily activities and social participation. The WG-SS intentionally moves away from diagnosis-based definitions and instead emphasizes the interaction between impairments and the environment, aligning with the International Classification of Functioning, Disability and Health (Madans et al., 2011).

The development of the WG-SS included extensive cognitive testing in multiple countries and languages. Through cognitive interviewing, researchers assessed how people understood the WG-SS items and response options (such as “some difficulty,” “a lot of difficulty,” and “cannot do at all”) and evaluated the cross-cultural applicability of the questions. These evaluations, conducted in collaboration with UNICEF and the National Center for Health Statistics (NCHS), affirmed that self-respondents were generally able to interpret the items as intended, though subtle differences emerged based on cultural context and expectations of “normal” functioning (Massey et al., 2014). Subsequent testing comparing parent-proxy responses to teen self-reports revealed that proxies often answered based on observable behaviors or third-party input, while self-respondents incorporated a broader range of subjective experiences in their answers (Massey et al., 2015).

While most early WG-SS cognitive testing focused on self-report, more recent studies have included proxy respondents and documented key differences in response patterns. For example, a 2020 cognitive testing study comparing the WG-SS and American Community Survey (ACS) disability questions found that when proxies answered for another household member, they relied heavily on observation and outside information (e.g., from teachers or doctors), rather than the target individual's self-perceived experience (Miller et al., 2021). In some cases, proxies were more likely to report limitations as severe, particularly when the limitation had observable effects on daily life or caregiving burden.

Despite these findings, many large-scale household surveys—including the National Health Interview Survey (NHIS)—continue to rely heavily on proxy reporting. In the NHIS, one adult household member (the Sample Adult) is selected to answer detailed health questions for themselves unless they are “physically or mentally unable to respond,” in which case a knowledgeable family member or caregiver may complete the interview as a proxy (National Center for Health Statistics, 2018). Although the Washington Group recommends self-response whenever feasible—emphasizing “for disability among adults, a self-respondent is preferred,” “the choice of respondent will impact the results,” and “in any analysis the type of respondent should be noted” (Washington Group on Disability Statistics, 2020, p. 6)—proxy use remains common in surveys like the NHIS due to logistical and practical constraints. This raises important questions about whether disability status is being measured consistently between self-respondents and proxy respondents. If proxies systematically differ in how they classify disability, this could affect the accuracy of prevalence estimates and have implications for health equity and policy.

### 1.1 Research purpose and hypothesis

The purpose of the current study is to examine whether individuals are differentially classified as having a disability under the Washington Group Short Set (WG-SS) depending on whether responses are provided by a proxy or self-reported. This study uses data from the National Health Interview Survey (NHIS) to evaluate whether proxy respondents are more or less likely to classify household members as having a disability under the WG-SS framework, even after accounting for sociodemographic characteristics; disability classification followed the recommendations for use provided by the Washington Group (Washington Group on Disability Statistics, 2020). This work aims to assess the response processes and person-centered accuracy of the WG-SS in the context of household surveys where proxy reporting is common.

We hypothesize that disability classification under the WG-SS is associated with reporting source, such that individuals reported on by a proxy will be classified differently—potentially more frequently—as having a disability compared to those who self-report. By distinguishing between the likelihood of disability identification, this study contributes to a more nuanced understanding of how proxy response influences disability classification and prevalence estimates in U.S. health surveillance.

## 2 Methods

### 2.1 Data

Data from the 2010 through 2018 National Health Interview Survey (NHIS) were acquired from IPUMS (Blewett et al., 2019). The total pooled sample size was 284,809, including both children and adults. Because the WG-SS questions are only administered to adults (18+), 144,925 cases for individuals under age 18 were removed. Of the people who were administered the WG-SS, 5,554 did not answer any of the six questions and were also removed from the sample. After removing these people, the sample size was 134,330. Following IPUMS guidance (https://nhis.ipums.org/nhis/userNotes_variance.shtml), the sample weight variable (SAMPWEIGHT) was divided by the number of years pooled together (nine) to produce weights representing the average U.S. population across the 9-year period.

### 2.2 WG-SS disability indicator

The dependent variable in the current study is whether a person is classified as having a disability based on their responses to the WG-SS items. Following the Washington Group's recommendations, if a person endorses “a lot of difficulty” or “cannot do at all,” they are considered as having a disability in the corresponding domain (Washington Group on Disability Statistics, 2020). People reporting at least one WG-SS disability are coded as 1; all others are coded as 0. Table 1 shows the six disability domains and the corresponding item on the survey.

**Table 1:** WG-SS items.

**Disability domain**	**Description**
Walking	How much difficulty do you walking or climbing stairs?
Remembering	How much difficulty do you have remembering or concentrating?
Communication	How much difficulty do you have communicating, for example understanding or being understood by others?
Selfcare	How much difficulty do you have with self-care, such as washing all over or dressing?
Hearing	How much difficulty do you have hearing even I using a hearing aid?
Vision	How much difficulty do you have seeing even if wearing glasses?

### 2.3 Proxy respondent

The independent variable of interest in the current study is whether the NHIS interview was completed by a proxy or not. This variable was created using two NHIS variables: (1) PROXYSA, which indicates whether a person used a proxy due to a physical or mental condition; and (2) SAPROXYAVAIL, which indicates whether a knowledgeable proxy was available to complete the interview. Respondents who did not use a proxy were coded as 0, while those who needed a proxy, and had one available, were coded as 1. In our sample, all respondents who needed a proxy had one available.

### 2.4 Sociodemographic variables

The sociodemographic variables used in the analysis were chosen because previous literature suggests they are associated with disability status, proxy respondent, or both. The included variables are as follows: age (18–85; Lauer et al., 2019; Mactaggart et al., 2016; Todorov and Kirchner, 2000), sex (0 = male, 1 = female; Elkasabi, 2021; Mactaggart et al., 2016; Lauer et al., 2019), marital status (0 = married, 1 = not married; Saito et al., 2024), race/ethnicity (0 = White, 1 = non-White; Lauer et al., 2019), Hispanic ethnicity (0 = no, 1 = yes; Lauer et al., 2019), education (0 = some college or more, 1 = high school or less; Elkasabi, 2021; Hall et al., 2022a; Shandra, 2018), employment status (0 = employed, 1 = not employed; Amilon et al., 2021; Hendershot, 2004; Shandra, 2018), health (0 = poor to 4 = excellent; Amilon et al., 2021; Amilon and Christensen, 2025; Li et al., 2015), and insurance coverage (0 = has coverage, 1 = no coverage; Kaye, 2019).

### 2.5 Analysis

A binary logistic regression was conducted to examine the relationship between proxy respondents (IV) and WG-SS disability status (DV) after controlling for sociodemographic characteristics. All analyses were conducted in Rstudio (Posit Team, 2024). The svyglm function in the survey package (Lumley, 2020) was used to conduct the logistic regression and to adjust for person weighting, stratification, and clustering due to the complex survey design of the NHIS. A likelihood-ratio test (LRT; Glover and Dixon, 2004), Wald test (Fox, 1997), and model accuracy (Baratloo et al., 2015), were used to assess model fit and performance. Listwise deletion was used for incomplete cases, resulting in a sample size of 133,025 people.

## 3 Results

### 3.1 Descriptive statistics

Using the WG-SS disability items, 10.9% of the sample reported having a disability. The majority of the sample were female, White/Caucasian, non-Hispanic, married, had some college education or more, were unemployed, and had health insurance coverage. The average age of the sample was 49 years (*SD* = 18.4), and the average self-reported health was 2.66 (SD = 1.08). A breakdown of disability domains and demographic characteristics by WG-SS disability indicator (described in Section 2.2) is presented in Tables 2, 3, respectively.

**Table 2:** Disability domain by WG-SS disability indicator^a^.

**Variable**	**No disability (*n*, %)**	**Disability (*n*, %)**	**Total^b^**
**Walking disability** ^c^			
No	119,651 (95.7)	5,439 (4.3)	125,090
Yes	0 (0)	9,240 (100)	9,240
**Remembering disability** ^d^			
No	119,651 (91.1)	11,632 (8.9)	131,283
Yes	0 (0)	3,047 (100)	3,047
**Communication disability** ^e^			
No	119,651 (89.8)	13,562 (10.2)	133,213
Yes	0 (0)	1,117 (100)	1,117
**Selfcare disability** ^f^			
No	119,651 (90)	13,229 (10)	132,880
Yes	0 (0)	1,450 (100)	1,450
**Hearing disability** ^g^			
No	119,651 (90.9)	11,931 (9.1)	131,582
Yes	0 (0)	2,748 (100)	2,748
**Vision disability** ^h^			
No	119,651 (90.8)	12,175 (9.2)	131,826
Yes	0 (0)	2,504 (100)	2,504

^a^Indicates a person reported “a lot of difficulty” or “cannot do at all” for at least one of the disability domains.

^b^n = 134,440.

^c^How much difficulty do you walking or climbing stairs?

^d^How much difficulty do you have remembering or concentrating?

^e^How much difficulty do you have communicating, for example understanding or being understood by others?

^f^How much difficulty do you have with self-care, such as washing all over or dressing?

^g^How much difficulty do you have hearing even I using a hearing aid?

^h^How much difficulty do you have seeing even if wearing glasses?

**Table 3:** Demographic characteristics by disability indicator^a^.

**Variable**	**No disability (*n*, %)**	**Disability (*n*, %)**	**Total^b^**
**Proxy respondent**			
No	118,533 (89.7)	13,592 (10.3)	132,125
Yes	887 (45.7)	1,053 (54.3)	1,940
Missing	231 (87.2)	34 (12.8)	265
**Sex**			
Male	54,677 (90.4)	5,802 (9.6)	60,479
Female	64,974 (88)	8,877 (12)	73,851
**Marital status**			
Not married	54,496 (91.9)	4,804 (8.1)	59,300
Married	65,155 (86.8)	9,875 (13.2)	75,030
**Race/ethnicity**			
White	92,969 (89.2)	11,307 (10.8)	104,276
Non-White	26,682 (88.8)	3,372 (11.2)	30,054
**Hispanic**			
No	101,183 (88.8)	12,785 (11.2)	113,968
Yes	18,468 (90.7)	1,894 (9.3)	20,362
**Education**			
Some college or more	75,544 (92.5)	6,161 (7.5)	81,705
High school or less	43,707 (83.9)	8,384 (16.1)	52,091
Missing	400 (74.9)	134 (25.1)	534
**Employment status**			
Not employed	74,944 (96.5)	2,749 (3.5)	77,693
Employed	44,640 (78.9)	11,927 (21.1)	56,567
Missing	67 (95.7)	3 (4.3)	70
**Insured**			
Insured	103,287 (88.4)	13,493 (11.6)	116,780
Not insured	15,948 (93.3)	1,152 (6.7)	17,100
Missing	416 (92.4)	34 (7.6)	450
**Average age (SD)**	48.27 (17.95)	62.29 (17.23)	49.81 (18.40)^d^
**Average health**^**c**^ **(SD)**	2.80 (0.99)	1.51 (1.11)	2.66 (1.08)^d^

^a^Indicates a person reported a disability in at least one of the disability domains.

^b^n = 134,440.

^c^Average for the self-reported health variable; ranges from 0 (poor) to 4 (excellent).

^d^Average (standard deviation) for the entire sample.

### 3.2 Binary logistic regression

A binary logistic regression was conducted to examine whether individuals were more likely to be classified as having a WG-SS disability when a proxy respondent completed the NHIS survey, controlling for sociodemographic characteristics. A likelihood ratio test (LRT) showed the full model fit significantly better than the null model, χ^2^(*df* = 9) = 24635.61, *p* < 0.001. A Wald test indicated the overall effect of the variables are statistically significant, χ^2^(*df* = 23) = 4912.50, *p* < 0.001. Using a threshold of model fitted values >0.50, participants were classified as having a WG-SS disability, otherwise no WG-SS disability, and model accuracy was computed. Model accuracy was calculated as the number of correctly identified cases divided by total cases. The model's accuracy was 90.35% (120,915/133,025), meaning the model correctly classified 90.35% of the cases. Proxy respondent was statistically significant, *exp*(β) = 4.48, *se* = 0.08, *p* < 0.001, 95% CI [3.86, 5.21], indicating proxy respondents were 4.48 times more likely to endorse a disability than self-report respondents. See Table 4 for the full regression table.

**Table 4:** Binary logistic regression predicting WG-SS disability indicator^a^.

**Variables**	***exp* (β)**	***se***	***t*-values**	***p*-value**	**95% Lower CI^b^**	**95% Upper CI^b^**
(Intercept)	0.22	0.05	−32.88	< 0.001	0.20	0.24
Age^c^	1.02	0.00	27.93	< 0.001	1.02	1.02
Female (reference group: male)	1.17	0.02	6.39	< 0.001	1.11	1.22
Not married (reference group: married)	1.50	0.02	16.87	< 0.001	1.43	1.57
Non-white (reference group: white)	0.83	0.03	−6.38	< 0.001	0.79	0.88
Hispanic ethnicity—yes (reference group: no)	0.87	0.04	−3.66	< 0.001	0.81	0.94
High school or less (reference group: some college or more)	1.24	0.03	8.61	< 0.001	1.18	1.31
Unemployed (reference group: employed)	2.69	0.03	34.20	< 0.001	2.54	2.85
Self-reported health^d^	0.39	0.01	−73.66	< 0.001	0.38	0.40
Not insured (reference group: insured)	0.87	0.05	−3.05	< 0.01	0.79	0.95
Proxy respondent—yes (reference group: proxy respondent—no)	4.48	0.08	19.52	< 0.001	3.86	5.21

^a^n = 133,025.

^b^Confidence intervals for the odds ratios.

^c^Age is mean centered, so the intercept represents average age (48.27).

^d^self-reported health variable; ranges from 0 (poor) to 4 (excellent).

## 4 Discussion

This study demonstrates that the likelihood of being classified as having a disability using the WG-SS is significantly associated with whether responses are provided by the individual themselves or by a proxy. Even after adjusting for sociodemographic characteristics, individuals with proxy respondents were 4.48 times more likely to be classified as disabled compared to those who self-reported. These findings raise important concerns about the comparability and construct validity of disability prevalence estimates derived from the WG-SS in household surveys where proxy reporting is common.

These results challenge a core assumption in the application of the WG-SS—that proxy and self-responses are interchangeable for the purpose of identifying disability. This introduces a new dimension to concerns about *validity by representation*: proxy respondents may be more likely to report observable limitations or apply more clinical or external thresholds, resulting in systematic differences in classification across respondent types.

An additional implication of these findings is the potential undercounting of disability among individuals who self-report. While proxy respondents may overreport observable difficulties, it is also possible—and perhaps more concerning—that self-respondents underreport functional limitations due to stigma, internalized ableism, lack of awareness, or differing interpretations of what constitutes “a lot of difficulty.” Research shows that stigma can discourage disclosure and lead to self-censorship regarding disability status (Bharadwaj et al., 2017; Prizeman et al., 2024). For instance, in Nepal, individuals often conceal disability in order to avoid social exclusion or to protect family reputation (Subedi, 2025). In the U.S., ASPE has noted that “proxy responses and stigma related to disability also might contribute to poor data quality” (Livermore et al., 2011). Still, the role of stigma in shaping proxy vs. self-reporting remains underexplored (Elkasabi, 2021). Thus, the increased likelihood of disability classification via proxy may reflect under-identification among self-respondents. Reducing the threshold for determining disability on the WG-SS to “some difficulty” would capture a larger proportion of people (Bourke et al., 2021) and could reduce discrepancies between proxy and self-reporters. However, adopting a lower threshold does not guarantee that individuals will identify as having a disability (Sakamoto and Kakuta, 2025), and it would limit comparability across reported results from other surveys since “a lot of difficulty” remains the most used threshold standard for disability determination; additional analyses would be needed to make those comparisons. Addressing this discrepancy requires not only improved survey design and validation processes but also supports to help self-respondents report functional limitations accurately (Hall et al., 2022b, 2025). Without such attention, efforts to measure disability prevalence may underestimate the true scope of disability in the population, with consequences for equity, resource allocation, and policy accountability.

These findings also suggest a potential gap in the cognitive testing and validation processes of the WG-SS. Although U.S.-based cognitive testing efforts—particularly those led by NCHS and Collaborating Center for Questionnaire Design and Evaluation Research (CCQDER)—did include proxy respondents, the primary focus was on ensuring comprehension and consistent interpretation of individual items. Much less attention has been paid to evaluating measurement equivalence across proxy and self-report contexts. This is a critical oversight given the widespread use of proxy reporting in large-scale federal surveys like the NHIS and American Community Survey, where proxy response is built into the data collection protocol.

In addition to our findings, prior research has consistently demonstrated that proxy respondents often differ from self-respondents in key sociodemographic characteristics, which may contribute to the observed disparities in disability reporting. For example, Todorov and Kirchner (2000), using data from the NHIS-D, found that proxies systematically underreported some disabilities (e.g., sensory and mental health limitations) for adults 18–64, while overreporting others, particularly activities of daily living (ADL) difficulties among older adults. Although their study did not use the WG-SS, it highlighted how differences between self- and proxy-reported disabilities can vary by the type and observability of the disability. In contrast, Elkasabi (2021), using the WG-SS, found that proxies were more likely to report observable disabilities than less visible ones, with differences linked to age, education, and gender between proxies and self-respondents. Future research should continue to explore the potential for residual confounding and examine how factors such as health literacy, language barriers, cultural norms, or differences in the proxy's relationship to the target respondent (e.g., spouse vs. adult child) could influence reporting patterns.

Overall, these findings highlight the need for caution when interpreting disability prevalence estimates derived from household surveys that use proxy respondents. Survey designers and policymakers should consider strategies to mitigate potential biases introduced by proxy reporting, including additional training for interviewers, validation studies comparing proxy and self-reports, and improved data collection protocols that prioritize self-response whenever possible.

### 4.1 Limitations

This study has several limitations that should be considered when interpreting the findings. First, although we examined the association between proxy response and disability classification using the WG-SS, the analysis was limited to a binary disability indicator (i.e., presence vs. absence of disability) rather than assessing the severity of functional limitations. Consequently, we cannot determine whether proxies differentially report the severity of disability across functional domains, which may be relevant for more nuanced analyses of disability experience. Second, the reasons why a proxy was used in the NHIS (i.e., physical or mental condition prohibiting self-response) are not fully captured in the analysis. Although the NHIS designates proxies only under specific conditions, in practice, there may be inconsistencies in how interviewers determine the need for a proxy or in how household members decide who responds and these factors could play a role in disability classification. Additionally, individuals were removed prior to analyses because they did not complete any of the WG-SS items and this could affect the representativeness of the sample. Finally, the cross-sectional design of the study precludes causal inference. While we found an association between proxy response and disability classification, we cannot conclude that proxy reporting itself causes differences in disability prevalence estimates.

### 4.2 Future research

Future studies should build on these findings by using designs and methods that directly test the impact of proxy response on disability classification. Experimental designs that randomly assign self-respondents to conditions with or without proxy reporting could help disentangle the effects of proxy use from characteristics of respondents who typically require a proxy. Applying differential item functioning (DIF) techniques would provide a more nuanced understanding of which WG-SS items are most sensitive to proxy vs. self-report differences, and which respondent or contextual factors drive those differences. Additional research should also explore how characteristics of the proxy (e.g., relationship to the respondent, caregiving role, health literacy) shape reporting patterns, to inform survey protocols and improve the validity of disability measurement.

### 4.3 Conclusion

The differences in proxy reporting are not merely technical. They have real implications for equity, access, and policy accountability. If proxy reporting systematically increases (or decreases) the likelihood of disability classification, prevalence estimates—and the programs or protections tied to those numbers—may be distorted. This is especially problematic when proxies are more likely to report for populations already at risk of under- or overrepresentation in disability data, such as older adults, people with cognitive disabilities, and children and adolescents.

In surveys where proxy reporting is used, the source of the response may influence whether someone is counted as having a disability. This finding has important implications for survey design, cognitive testing, and the interpretation of disability data. We recommend that future validation studies of the WG-SS—and disability measurement tools more broadly—treat reporting source not as a procedural footnote, but as a meaningful source of variation with implication for data quality, equity, and the validity of disability statistics.

## Data Availability

Publicly available datasets were analyzed in this study. This data can be found at: https://nhis.ipums.org/nhis/.

## References

[B1] Amilon A. Christensen M. L. (2025). Changing disability status and changes in health, participation restrictions and activity limitations in Denmark: does the choice of measure matter? Scand. J. of Disabil. Res. 27, 15–29. 10.16993/sjdr.1191

[B2] Amilon A. Hansen K. M. Kjær A. A. Steffensen T. (2021). Estimating disability prevalence and disability-related inequalities: does the choice of measure matter? Soc. Sci. Med. 272:113740. 10.1016/j.socscimed.2021.113740 33571943

[B3] Baratloo A. Hosseini M. Negida A. El Ashal G. (2015). Part 1: simple definition and calculation of accuracy, sensitivity and specificity. Emergency 3, 48–49. 26495380 PMC4614595

[B4] Bharadwaj P. Pai M. M. Suziedelyte A. (2017). Mental health stigma. Econ. Lett. 159, 57–60. 10.1016/j.econlet.2017.06.028

[B5] Blewett L. A. Rivera Drew J. A. King M. L. Williams K. C. Del Ponte N. Convey P. (2019). IPUMS Health Surveys: National Health Interview Survey, Version 7.4 [Dataset]. Minneapolis, MN: IPUMS.

[B6] Bourke J. A. Nichols-Dunsmuir A. Begg A. Dong H. Schluter P. J. (2021). Measuring disability: an agreement study between two disability measures. Disabil. Health J. 14:100995. 10.1016/j.dhjo.2020.100995 32972900

[B7] Elkasabi M. (2021). Differences in proxy-reported and self-reported disability in the demographic and health surveys. J. Surv. Stat. Methodol. 9, 335–351. 10.1093/jssam/smaa041

[B8] Fox J. (1997). Applied Regression Analysis, Linear Models, and Related Methods. Thousand Oaks, CA: SAGE Publications, Inc.

[B9] Glover S. Dixon P. (2004). Likelihood ratios: a simple and flexible statistic for empirical psychologists. Psychon. Bull. Rev. 11, 791–806. 10.3758/BF03196706 15732688

[B10] Hall J. P. Kurth N. K. Goddard K. S. (2022a). Assessing factors associated with social connectedness in adults with mobility disabilities. Disabil. Health J. 15:101206. 10.1016/j.dhjo.2021.101206 34489203

[B11] Hall J. P. Kurth N. K. Ipsen C. Myers A. Goddard K. (2022b). Comparing measures of functional difficulty with self-identified disability: implications for health policy. Health Aff. 41, 1433–1441. 10.1377/hlthaff.2022.00395 36190890 PMC10353341

[B12] Hall J. P. Thomas K. McCormick B. Kurth N. K. (2025). Undercounts of people with serious mental illness using the washington group short set. Front. Psychiatry 16:1606154. 10.3389/fpsyt.2025.1606154 40636437 PMC12238763

[B13] Hendershot G. E. (2004). The effects of survey nonresponse and proxy response on measures of employment for persons with disabilities. Disabil. Stud. Q. 24. 10.18061/dsq.v24i2.481

[B14] Kaye H. S. (2019). Disability-related disparities in access to health care before (2008–2010) and after (2015–2017) the Affordable Care Act. Am. J. Public Health, 109, 1015–1021. 10.2105/AJPH.2019.305056 31095413 PMC6603452

[B15] Lauer E. A. Henly M. Coleman R. (2019). Comparing estimates of disability prevalence using federal and international disability measures in national surveillance. Disabil. Health J. 12, 195–202. 10.1016/j.dhjo.2018.08.008 30268508

[B16] Li M. Harris I. Lu Z. K. (2015). Differences in proxy-reported and patient-reported outcomes: assessing health and functional status among Medicare beneficiaries. BMC Med. Res. Methodol. 15, 1–10. 10.1186/s12874-015-0053-7 26264727 PMC4534114

[B17] Livermore G. Whalen D. Stapleton D. C. (2011). Assessing the need for a national disability survey (No. 4722b35937804264aed3fcbaf7fd1514). Math. Policy Res.

[B18] Lumley T. (2020). Survey: Analysis of Complex Survey Samples (Version 4.1) [R Package].

[B19] Mactaggart I. Kuper H. Murthy G. V. S. Oye J. Polack S. (2016). Measuring disability in population based surveys: the interrelationship between clinical impairments and reported functional limitations in Cameroon and India. PLoS One 11:e0164470. 10.1371/journal.pone.0164470 27741320 PMC5065175

[B20] Madans J. H. Loeb M. E. Altman B. M. (2011). Measuring disability and monitoring the UN Convention on the Rights of Persons with Disabilities: the work of the Washington Group on Disability Statistics. BMC Public Health 11. 10.1186/1471-2458-11-S4-S4 21624190 PMC3104217

[B21] Massey M. Chepp V. Zablotsky B. Creamer C. (2014). Analysis of Cognitive Interview Testing of Child Disability Questions in Five Countries. Hyattsville, MD: National Center for Health Statistics. Available online at: https://wwwn.cdc.gov/qbank/report/Massey_NCHS_2014_UNICEF_Child_Disability.pdf (Accessed June 1, 2025).

[B22] Massey M. Scanlon P. Lessem S. Cortes L. Villarroel M. Salvaggio M. (2015). Analysis of Cognitive Testing of Child Disability Questions: Parent-Proxy vs. Teen Self-Report. Hyattsville, MD: National Center for Health Statistics. Available online at: https://wwwn.cdc.gov/qbank/report/Meredith_2016_NCHS_MCFD.pdf (Accessed June 2, 2025).

[B23] Miller K. Vickers J. B. Scanlon P. (2021). Comparison of American Community Survey and Washington Group Disability Questions. Hyattsville, MD: National Center for Health Statistics. Available online at: https://wwwn.cdc.gov/QBank/Search/ReportDetails.aspx (Accessed June 5, 2025).

[B24] National Center for Health Statistics Z. Z.. (2018). 2018 CAPI Manual for NHIS Field Representatives. U.S. Department of Health and Human Services, Centers for Disease Control and Prevention. Available online at: https://ftp.cdc.gov/pub/Health_Statistics/NCHS/Survey_Questionnaires/NHIS/2018/frmanual.pdf (Accessed June 7, 2025).

[B25] Posit Team Z. Z. (2024). RStudio: Integrated Development Environment for R. Boston, MA: Posit Software. Available online at: http://www.posit.co/ (Accessed June 9, 2025).

[B26] Prizeman K. McCabe C. Weinstein N. (2024). Stigma and its impact on disclosure and mental health secrecy in young people with clinical depression symptoms: a qualitative analysis. PLoS ONE 19:e0296221. 10.1371/journal.pone.0296221 38180968 PMC10769096

[B27] Saito T. Imahashi K. Yamaki C. (2024). The first use of the Washington Group Short Set in a national survey of Japan: characteristics of the new disability measure in comparison to an existing disability measure. Int. J. Environ. Res. Public Health 21:1643. 10.3390/ijerph21121643 39767482 PMC11675656

[B28] Sakamoto M. Kakuta N. (2025). Bayesian modeling of underreported disabilities: gender insights from the Bangladesh national household survey. Disabil. Health J. 101922. 10.1016/j.dhjo.2025.101922 40634166

[B29] Shandra C. L. (2018). Disability as inequality: social disparities, health disparities, and participation in daily activities. Soc. Forces 97, 157–192. 10.1093/sf/soy031

[B30] Subedi T. N. (2025). Stigmatization of people with disability in Nepal. SMC J. Sociol. 2, 27–47. 10.3126/sjs.v2i2.74839

[B31] Todorov A. Kirchner C. (2000). Bias in proxies' reports of disability: data from the National Health Interview Survey on disability. Am. J. Public Health 90, 1248–1253. 10.2105/AJPH.90.8.1248 10937005 PMC1446336

[B32] Washington Group on Disability Statistics Z. Z. (2020). The Data Collection Tools Developed by the Washington Group on Disability Statistics and their Recommended Use. Available online at: https://www.washingtongroup-disability.com/fileadmin/uploads/wg/Documents/WG_Implementation_Document__1_-_Data_Collection_Tools_Developed_by_the_Washington_Group.pdf (Accessed June 3, 2025).

